# PD-L1 Mediates IFNγ-Regulation of Glucose but Not of Tryptophan Metabolism in Clear Cell Renal Cell Carcinoma

**DOI:** 10.3389/fonc.2022.858379

**Published:** 2022-05-17

**Authors:** Mamatha Garige, Susmita Ghosh, Alexis Norris, Guangyuan Li, Sarah Poncet, Chao-Kai Chou, Wells W. Wu, Rong-Fong Shen, Carole Sourbier

**Affiliations:** ^1^ Division of Biotechnology Review and Research 1, Office of Biotechnology Products, Office of Pharmaceutical Quality, Center for Drug Evaluation and Research, United States Food and Drug Administration, Silver Spring, MD, United States; ^2^ Division of Animal Bioengineering and Cellular Therapies, Office of New Animal Drug Evaluation, Center for Veterinary Medicine, United States Food and Drug Administration, Rockville, MD, United States; ^3^ Facility for Biotechnology Resources, Center for Biologicals Evaluation and Research, United States Food and Drug Administration, Silver Spring, MD, United States

**Keywords:** PD-L1 (B7-H1, CD274), clear cell renal cell carcinoma (ccRCC), interferon gamma, metabolism, tryptophan, kynurenine, glycolysis

## Abstract

The immune checkpoint programmed death-ligand 1 (PD-L1) is expressed on the cell surface of tumor cells and is key for maintaining an immunosuppressive microenvironment through its interaction with the programmed death 1 (PD-1). Clear cell renal cell carcinoma (ccRCC) is a highly immunogenic cancer characterized by an aberrant aerobic glycolytic metabolism and is known to overexpress PD-L1. Multiple immunotherapies have been approved for the treatment of ccRCC, including cytokines and immune checkpoint inhibitors. Recently the intrinsic role of PD-L1 and interferon gamma (IFNγ) signaling have been studied in several types of tumor cells, yet it remains unclear how they affect the metabolism and signaling pathways of ccRCC. Using metabolomics, metabolic assays and RNAseq, we showed that IFNγ enhanced aerobic glycolysis and tryptophan metabolism in ccRCC cells *in vitro* and induced the transcriptional expression of signaling pathways related to inflammation, cell proliferation and cellular energetics. These metabolic and transcriptional effects were partially reversed following transient PD-L1 silencing. Aerobic glycolysis, as well as signaling pathways related to inflammation, were not induced by IFNγ when PD-L1 was silenced, however, tryptophan metabolism and activation of Jak2 and STAT1 were maintained. Our data demonstrate that PD-L1 expression is required to mediate some of IFNγ’s effect in ccRCC cells and highlight the importance of PD-L1 signaling in regulating the metabolism of ccRCC cells in response to inflammatory signals.

## Introduction

With 73,750 patients newly diagnosed in the US in 2020 and 14,830 deaths, kidney cancer is one of the most lethal urologic cancers (1). Clear cell renal cell carcinoma (ccRCC) is the most common type of kidney tumors and represents over 75% of all cases (2). In 90% of ccRCC cases, the tumor suppressor gene von Hippel Lindau (*VHL*) is mutated or methylated which leads to the disruption of the VHL complex and induces a “pseudo-hypoxic” environment (3–5). The VHL protein is part of an oxygen-sensing complex that recognized hydroxylated hypoxia inducible factor (HIF) and targets it for ubiquitin degradation (6, 7). Under hypoxia, HIF is not hydroxylated, and therefore not recognized by the VHL complex, leading to its stabilization and activation. In ccRCC, VHL mutation/methylation leads to the aberrant activation of HIF signaling pathway resulting in an increased aerobic glycolysis and angiogenesis. A multi-omic study by the Cancer Genome Atlas reported that the phenotype of advanced ccRCC was consistent with a metabolic shift toward aerobic glycolysis with grade and decreased oxidative phosphorylation (8). This suggests that the adaptation of ccRCC’s metabolism may be critical for growth and survival. In addition, ccRCC is considered immunogenic (9). It contains tumor-infiltrating immune cells, including macrophages and lymphocytes (10). While infiltered lymphocytes could elicit an immune reaction to eliminate cancer cells and improve prognosis in several cancers (11), in ccRCC, high immune infiltration correlated with poor prognosis and reduced survival of ccRCC patients (12).

The tumor microenvironment (TME) is a highly complex mix of extracellular matrix, stromal cells, immune cells, and cancer cells (13). Innate and adaptive immune cells such as activated cytotoxic CD8+ T lymphocytes cells and natural killer cells produce interferon gamma (IFNγ) in the tumor microenvironment (14), which in turn induces the expression of the programmed death ligand 1 (PD-L1 or B7-H1) on tumor cells (15). PD-L1 or B7-H1 is an immunosuppressive cell surface protein that interacts with the programmed death 1 (PD-1), a cell surface protein expressed on activated immune cells. The interaction of PD-1 and PD-L1 plays an important role in the establishment of an immunosuppressive TME that supports tumor growth. This serves as a basis of the mechanism of action of immune checkpoint inhibitors (ICIs) that block the interaction of PD-1 with PD-L1, thus reducing or eliminating immunosuppressive signals and could lead to enhanced immune cell activation and enhanced antitumor immunity (16–19).

The intrinsic role of PD-L1 on tumor cells has been studied in several cancer types and has been linked to tumor growth through its involvement in multiple signaling pathways, including cellular metabolism (20–25). In contrast, IFNγ could have contrasting functions depending on the targeted cells and the composition of the TME, having either an antitumoral effect or a tumorigenic effect. For example, IFNγ could have an anti-proliferative effect and induces apoptosis *via* the JAK/STAT pathway (26), while it could also induce the expression of PD-L1 and of indoleamine-2,3-dioxygenase (IDO) (27, 28). IDO is a key enzyme in the tryptophan metabolism pathway and catalyzes the degradation of tryptophan and the generation of kynurenine. It is involved in cancer growth and therapeutic resistance (29–31). Recent studies have demonstrated that most tumor types, including kidney tumors, express IDO (32). In colon cancer, kynurenine has been described as an oncometabolite and is necessary to maintain continuous colon cancer cells proliferation and to cause T-cell inactivation in the TME, leading to immune evasion and survival of cancer cells (33). In ccRCC, the activation of the kynurenine pathway has been associated with poor outcome (34).

Because advanced ccRCC display both aberrant metabolism and elevated immune infiltration, we investigated whether intrinsic IFNγ signaling and PD-L1 could affect the metabolism of ccRCC. Understanding how IFNγ and the microenvironment affect ccRCC metabolism and signaling pathways may provide the necessary knowledge for the development of biomarkers of efficacy for therapies modulating the TME, such as immunotherapies.

## Materials and Methods

### Cell Culture and Generation of Stable Cell Lines

The ccRCC cell lines 786-O and A498 were purchased from ATCC. They were cultured in Dulbecco’s Modified Eagle’s Medium (DMEM) growth media containing 25mM glucose and glutamine supplemented with 10% heat-inactivated fetal bovine serum and 1% penicillin-streptomycin at 37°C in a humidified atmosphere of 5% CO2.

Stable 786-O and A498 cell lines expressing Renilla luciferase were generated by transducing cells with LP462-025 lentiviral particles (Genecopia). Briefly, 786-O and A498 cells were transduced with lentiviral particles at a MOI (multiplicity of infection) of 5 in complete media with polybrene at a concentration of 5 μg/mL. Transduced cells were selected with media containing puromycin at a concentration of 4 μg/mL. Renilla Luciferase activity in cells was measured according to the manufacturer protocol.

CD8+ Cytotoxic lymphocytes (CTLs) were purchased from Stem Cell Technologies. They were cultured in RPMI supplemented with 10% FBS, 1% penicillin and streptomycin and 0.1 mg/mL hIL2. They were activated with ImmunoCult™ Human CD3/CD28 T cell Activator (Stem Cell Technologies) at a concentration of 25 μL/mL of cell suspension.

### siRNA Transfection

On-Target plus SMARTpool small interfering RNAs (siRNAs) against PD-L1 was purchased from Dharmacon. All siRNA transfections were conducted at a final concentration of 20 nM using Lipofectamine RNAiMAX (Invitrogen) following the manufacturer instructions. Non-targeting pool siRNA was used as a control (Cnsi). Transient silencing experiments had four groups, Cnsi (control scrambled siRNA, untreated), Cnsi IFNγ (IFNγ treated), PD-L1si (PD-L1 siRNA) and PD-L1si IFNγ (PD-L1 siRNA and IFNγ treatment). A498 and 786-O cells were transfected with control (scrambled siRNA) or PD-L1 siRNA, at the end of 24 hours of transfection, select groups were treated with IFNγ (50 ng/mL) for another 24 hours. At the end of the treatment period, cells were utilized for either Western blotting, RNA extraction, or co-culture experiments.

### Co-Culture of ccRCC Cells and Cytotoxic Lymphocytes

For co-culture experiments, silencing of PD-L1 was carried out with Renilla luciferase stable transfected A498 and 786-O cells in 48-well plates as described in the above section. At the end of silencing experiment, cell culture media was removed, and cells were washed with complete DMEM and co-cultured with CTLs at a ratio of 1:3 for a period of 24 hours. The number of live A498 and 786-O cells was assessed by measuring Renilla luciferase using a plate reader.

786-O and A498 stable cells lines expressing Renilla luciferase were plated in 48-well plates at a density of 1.5x10^4^ cells per well. The next day, cells were treated with IFNγ at a concentration of 50 ng/mL for 24 hours. At the end of the treatment period, culture media was removed, and fresh media was added. Appropriate groups were treated with avelumab, durvalumab and atezolizumab at a concentration of 20 μg/mL for 2 hours prior to co-culturing with CTLs. CTLs were added at an optimized ratio of 1:3 and incubated for 24 hours for A498 cells and 48 hours for 786-O cells. At the end of the incubation period, the number of live 786-O and A498 cells was assessed by measuring Renilla luciferase activity; and apoptosis was assessed by measuring caspase 3/7 activity using a Caspase-Glo 3/7 assay kit (Promega).

### Western Blots

Whole-cell lysates of A498, 786-O or CTLs isolated from co-culture experiments were prepared using radioimmunoprecipitation assay (RIPA) buffer containing protease inhibitor cocktail (Roche). The protein concentration was quantified using a BCA protein assay kit (ThermoScientific) and twenty microgram of total protein per lane was used for immunoblotting. The blots were probed with primary antibodies against PD-L1 (Abcam), PD-L2 (Abcam) and phospho-MTOR, MTOR, phospho-p70S6K, p70S6k, phosphor-fructokinase, hexokinase, pyruvate kinase, phospho-LDHA, LDHA, pyruvate dehydrogenase and beta-actin (all from Cell Signaling). Immunoreactivity was revealed with an ECL-Plus kit (ThermoScientific) using the ChemiDoc MP system (Bio-Rad).

### Oxygen Consumption Rates and Extracellular Acidification Rates

Metabolism of A498 and 786-O cells was assessed using the Seahorse XF96 Analyzer (Agilent Seahorse Bioscience, CA). A Seahorse XF Sensor Cartridge was hydrated the day before running the XF Assay in 96-well XF Utility Plate with 200 μL of Seahorse XF Calibrant Solution per well. The hydrated cartridge was kept in a non-CO_2_ 37°C incubator for 24 hours. On the day of analysis, the cells were washed three times with unbuffered XF Assay Media supplemented with 10 mM glucose, 2 mM sodium pyruvate, and 2 mM glutamine (adjusted to pH 7.4) and incubated in non-CO2 37°C incubator for one hour prior to performing the analysis in the analyzer.

### Glucose and Glutamine Uptake Analysis

Glucose and glutamine content in the cell culture media was measured utilizing Glucose-Glo™ and Glutamine/Glutamate-Glo™ Assay kits (Promega, WI) respectively following the manufacturer’s instructions. Glucose and glutamine were measured in the cell culture media, which gives an indirect measure of the amount of glucose and glutamine consumed by cells. The values were normalized to media without cells treated in the same way as test samples.

### Measurement of Metabolites

Metabolites were extracted and analyzed at HMT Metabolomics (Boston, MA). Briefly, two groups, control and IFNγ-treated groups, for each A498 and 786-O cell line were cultured in 10-cm culture dishes in triplicate. Both cells were plated at a density of 2.5x10^6^ per culture dish, and after culturing for 24 hours, cells were with IFNγ at a concentration of 50 ng/mL for 24 hours. At the end of the treatment period, cells were collected and counted before being washed twice with 5% mannitol solution (10 mL first and then 2 mL). Cells were then pelleted, stored at -80°C, and shipped to HMT Metabolomics for metabolite extraction and analysis. The cells were then treated with 800 µL of methanol and left at rest for 30 seconds to inactivate enzymes. The cell extract was treated with 550 µL of Milli-Q water containing internal standards (H3304-1002, Human Metabolome Technologies, Inc., Tsuruoka, Japan) and left at rest for another 30 seconds. The extract was obtained and centrifuged at 2,300×g and 4°C for 5 min and then 800 µL of upper aqueous layer was centrifugally filtered through a Millipore 5-kDa cutoff filter at 9,100×g and 4°C for 120 minutes to remove proteins. Metabolite measurements were normalized to cell count. The filtrate was centrifugally concentrated and re-suspended in 50 µL of Milli-Q water for CE-MS analysis. Cationic compounds were measured in the positive mode of CE-TOFMS, and anionic compounds were measured in the positive and negative modes of CE-MS/MS according to the methods developed by Soga, et al. (35). Peaks detected by CE-TOFMS and CE-MS/MS were extracted using automatic integration software (MasterHands, Keio University, Tsuruoka, Japan and MassHunter Quantitative Analysis B.04.00, Agilent Technologies, Santa Clara, CA, USA, respectively) to obtain peak information, including m/z, migration time (MT), and peak area. The peaks were annotated with putative metabolites from the HMT metabolite database based on their MTs in CE and m/z values determined by TOFMS. The tolerance range for the peak annotation was configured at ±0.5 minutes for MT and ±10 ppm for m/z. In addition, concentrations of metabolites were calculated by normalizing the peak area of each metabolite with respect to the area of the internal standard and by using standard curves, which were obtained by three-point calibrations.

### RNA Extraction and RNA-Sequencing

Total RNA from three groups (Cnsi, Cnsi-IFNγ, and PD-L1si IFNγ) for both A498 and 786-O cell lines, was extracted using RNeasy Mini Kit (Qiagen, Germany) according to the manufacturer’s protocol. Quality of the RNA samples was checked using the Agilent Bioanalyzer RNA Nano Chip (Santa Clara, CA) to determine RNA Integrity Number (RIN) ≥ 8. RNA samples were processed following the Illumina (San Diego, CA) TruSeq Stranded messenger RNA Sample Preparation Kit performed at FDA Core Facility (36). Briefly, poly (A) tailed RNA was purified from 500 ng of total RNA, fragmented, and reverse-transcribed into cDNAs. Double strand cDNAs were adenylated at the 3’ ends and individually indexed, followed by limited-cycle (15) amplification. Paired-end sequencing (100x2 cycles) of multiplexed mRNA samples was carried out on Illumina NovaSeq 6000 (A498) and NExtSeq 500 (786-O) sequencers. Sequencing reads raw data were converted to fastq files by bcl2fastq2 program (version 2.19.0) for subsequent data analysis. Raw fastq files and processed gene counts are available from NCBI GEO (Accession #GSE199107).

### Differential Gene Expression and Geneset Enrichment Analysis

Sequencing reads were trimmed using trimmomatic (version 0.36.6; parameters: SLIDINGWINDOW:4:20 MINLEN:50) and then aligned to GRCh38/hg38 using HISAT2 (version 2.1.0; parameters: –n-ceil L,0.0,0.15 –mp 6,2 –no-softclip –np 1 –rdg 5,3 –rfg 5,3 –sp 2,1 –score-min L,0.0, -0.2 –pen-cansplice 0 –pen-noncansplice 12 –pen-canintronlen G,-8.0,1.0 –pen-noncanintronlen G,-8.0,1.0 –min-intronlen 20 –max-intronlen 500000). Gene counts were estimated using featureCounts (version 1.6.3; parameters: -s 1 -t ‘exon’ -g ‘gene_id’ -J -Q 12 –minOverlap 1 –fracOverlap 0 –fracOverlapFeature 0) with GENCODE gene annotation (version 33; Ensembl version 99). Protein-coding genes, with at least 10 counts in at least one group, were normalized using the TMM method and subjected to differential expression testing using Quasi-Likelihood F-Test in edgeR R package using robust settings (version 3.28.1; **Supplemental Tables 1**, **2**). Geneset enrichment analysis (GSEA) was then performed on the differential gene expression results using the fgsea method in the clusterProfiler R package (version 3.14.3; 1000 permutations) for the following genesets: KEGG and MSig hallmark pathways. Genesets with 10-500 genes were tested and considered significantly enriched when FDR < 0.05 by BH method. Statistical analyses were performed using R version 3.6.3.

### Data Analysis

Statistical analysis was performed by Prism 5.0 (GraphPad Software, Inc., San Diego, CA) with two-tailed Student’s t-test. A p-value below 0.05 was considered statistically significant. Data are presented as mean ± SD.

## Results

### IFNγ Increases Aerobic Glycolysis and Tryptophan Metabolism in A498 and 786-O ccRCC Cells

**Figure 1:**
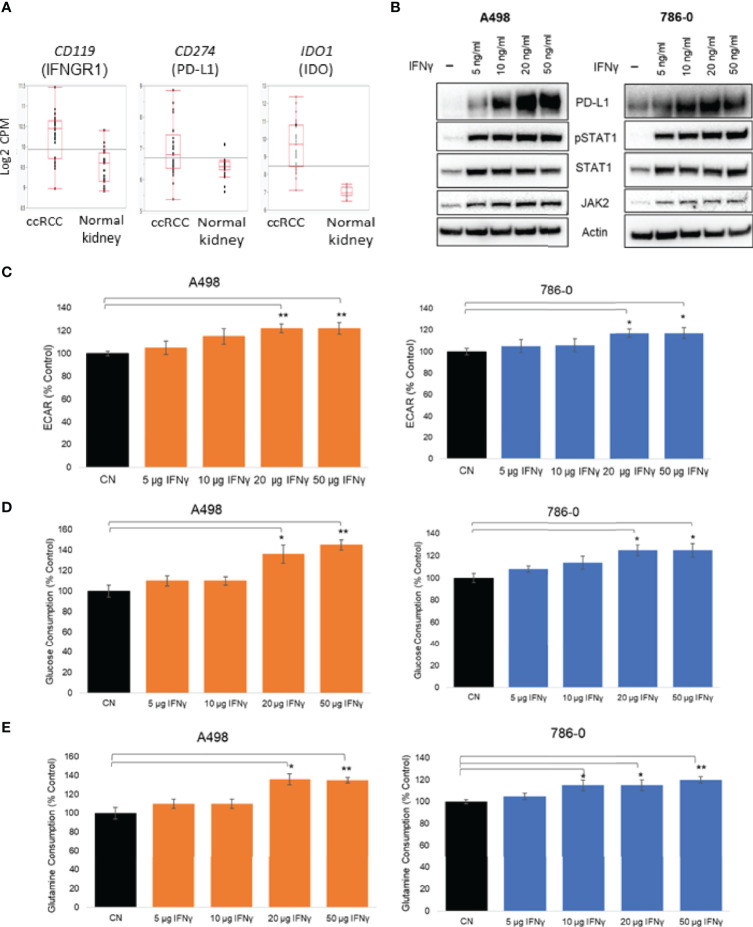
Effect of IFNγ treatment on A498 and 786-O ccRCC cells metabolism. **(A)** Expression of *CD119*, *CD274* and *IDO1* in ccRCC compared to normal kidney; **(B)** Representative immunoblot of PD-L1, PD-L2, JAK2 and pSTAT1 in A498 and 786-O cells treated with IFNγ (5, 10, 20 and 50 ng/mL) for 24 hours; **(C)** A surrogate for lactate secretion (extra cellular acidification rates, ECAR) and oxygen consumption rates (OCR) were measured using a bioanalyzer following IFNγ treatment (5, 10, 20 and 50 ng/mL for 24 hours) in A498 and 786-O cells; Glucose **(D)** and glutamine **(E)** consumptions were measured following IFNγ treatment (5, 10, 20 and 50 ng/mL for 24 hours)The data are presented as mean ± SD (n=3) *p<0.05; **p<0.01.

Upon exposure to IFNγ present in the microenvironment, cancer cells increase the expression of PD-L1, which interacts with PD-1 expressed on T cells and inhibits the antitumor immune response (19). We looked at the mRNA expression of key players of the IFNγ signaling in a publicly available dataset of ccRCC specimens (GSE36895 (37). As shown in **Figure 1A**, the expression of IFNγ signaling pathway members PD-L1, IFNGR1 and IDO1 were upregulated in ccRCC compared to normal tissues. The exposure of ccRCC cells to IFNγ was recapitulated *in vitro* by treating two ccRCC cell lines, A498 and 786-O, with a range of doses of IFNγ (5 to 50 ng/mL)for 24 hours and assessing protein expression by immunoblotting. IFNγ exposure increased the expression of PD-L1 in both A498 and 786-O cell lines in a dose concentration manner, as well as the protein expression of Jak2 and STAT1 and the phosphorylation status of STAT1 (**Figure 1B** and **Supplemental Figure 1A**), reflecting that the IFNγ pathway was activated. Because both JAK2 and PD-L1 have been reported to regulate metabolic processes including glucose metabolism (20, 25, 38), we investigated whether IFNγ influenced the metabolism of ccRCC cells using a Seahorse bioanalyzer (Agilent). IFNγ treatment increased the extracellular acidification rate (ECAR) of both A498 and 786-O cell lines (**Figure 1C**) without affecting their oxygen consumption rate (OCR) (**Supplemental Figure 1B**), suggesting that IFNγ signaling supports the shift of ccRCC cells towards aerobic glycolysis. Glucose and glutamine consumption were also increased following IFNγ treatment (**Figures 1D, E** respectively).

**Figure 2:**
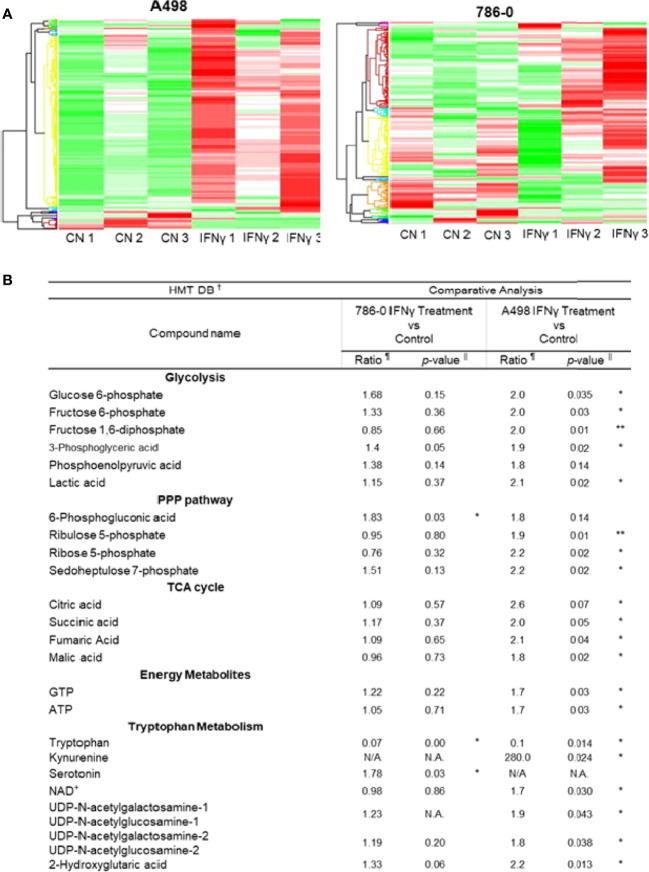
IFNγ treatment promotes aerobic glycolysis and tryptophane metabolism in A498 and 786-O. **(A)** The heat maps indicate a distinct metabolic rewiring following IFNγ treatment in A498 and 786-O cells as compared to controls. n=3 for each group. **(B)** Comparative analysis of metabolites of glycolysis, PPP (pentose phosphate pathway), TCA cycle (tricarboxylic acid), and energy metabolites between IFNγ treated and control A498 and 786-O cells. Putative metabolites which were assigned on the basis of m/z and MT in HMT standard compound library. The ratio is computed by using averaged detection values. The latter was used as denominator. The p-value is computed by Welch’s t-test (*p<0.05, **p<0.001). N.A., not applicable.

To further understand the metabolic effect of IFNγ in ccRCC cells, we performed metabolomic studies on both A498 and 786-O cell lines (**Figure 2A**, IFNγ at 50 ng/mL). Metabolites associated with glycolysis, the pentose-phosphate pathway and the TCA cycle, as well as ATP and GTP were significantly increased following IFNγ treatment in A498 cells, and there was a trend increasing in 786-O (**Figure 2B**) supporting the idea that IFNγ increased the glycolytic energy influx and the TCA cycle which resulted in an increased ATP and GTP production in both A498 and 786-O cell lines. Since IDO expression is regulated by IFNγ and is involved in tryptophan metabolism by converting tryptophan into kynurenine, we looked at the effect of IFNγ treatment on metabolites from the tryptophan pathway. Tryptophan was decreased, while kynurenine, serotonin, and NAD^+^ were increased in the cells treated with IFNγ compared to untreated cells (**Figure 2B**). These data indicate that IFNγ promotes the metabolism of tryptophan in 786-O and A498 cells. The hexosamine pathway was also enhanced by IFNγ treatment, as indicated by the increased in UDP-N-acetylglucosamine (UDP-GlcNA), which led to an increase in protein glycosylation.

These data demonstrate that the exposure of ccRCC cells to IFNγ had a global effect on ccRCC cells’ metabolism, suggesting that IFNγ may support the aberrant metabolic shift of ccRCC towards aerobic glycolysis, while also modulating tryptophan metabolism and NAD^+^ homeostasis.

### Blocking the Inducible Expression of PD-L1 Partially Reverses the Metabolic and Inflammatory Effects of IFNγ in A498 and 786-O Cells

**Figure 3:**
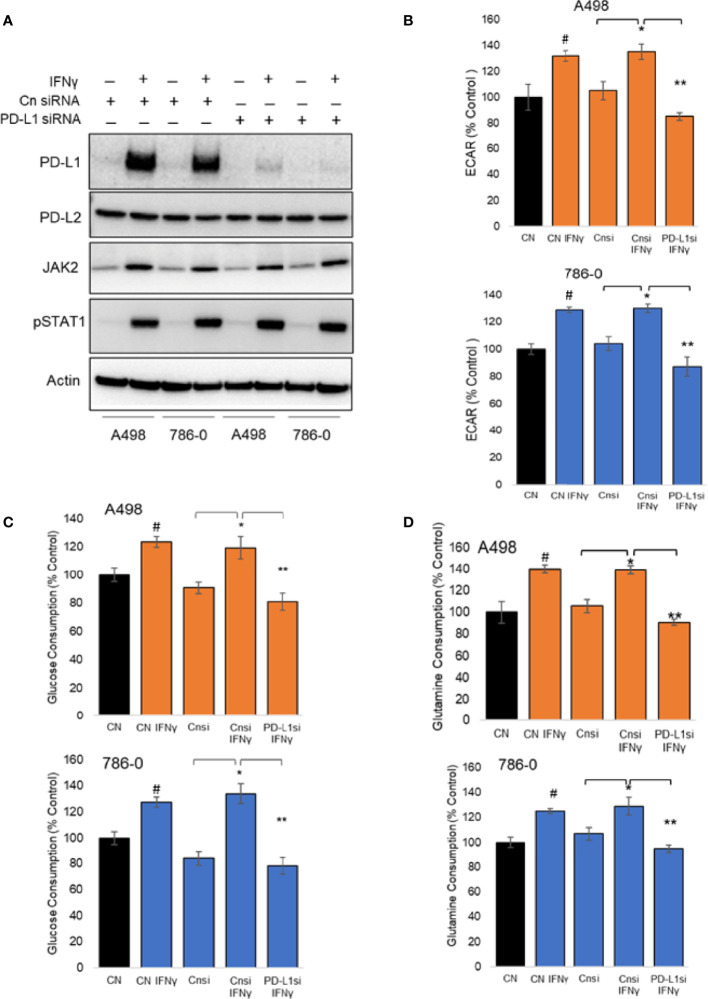
Metabolic effect of transient silencing of inducible expression of PD-L1 in A498 and 786-O cells. **(A)** 24 hours after silencing of PD-L1 (PD-L1 siRNA) or scrambled siRNA used as controls (Cn siRNA), A498 and 786-O cells were treated with IFNγ (50 ng/ml) for 24 hours, n=3. The protein expression of PD-L1, PD-L2, JAK2, STAT1 and the phosphorylation of STAT1 was then assessed by immunoblotting. **(B)** the metabolic effect of PD-L1 silencing was assessed using a bioanalyzer in IFNγ treated and PD-L1 silenced A498 and 786-O cells. A498 and 786-O cells without IFNγ treatment are used as controls. ECAR and OCR data are presented as mean ± SD (n=3) *p<0.05, **p<0.001. **(C, D)** A decrease in glucose and glutamine consumption was observed in PD-L1 silenced (PD-L1si IFNγ) A498 and 786-O cells as compared to IFNγ treated (Cnsi IFNγ) treated cells. The data are presented as mean ± SD (n=3) *p<0.05, **p<0.001, ^#^p<0.05 for CN IFNγ compared to CN.

**Figure 4:**
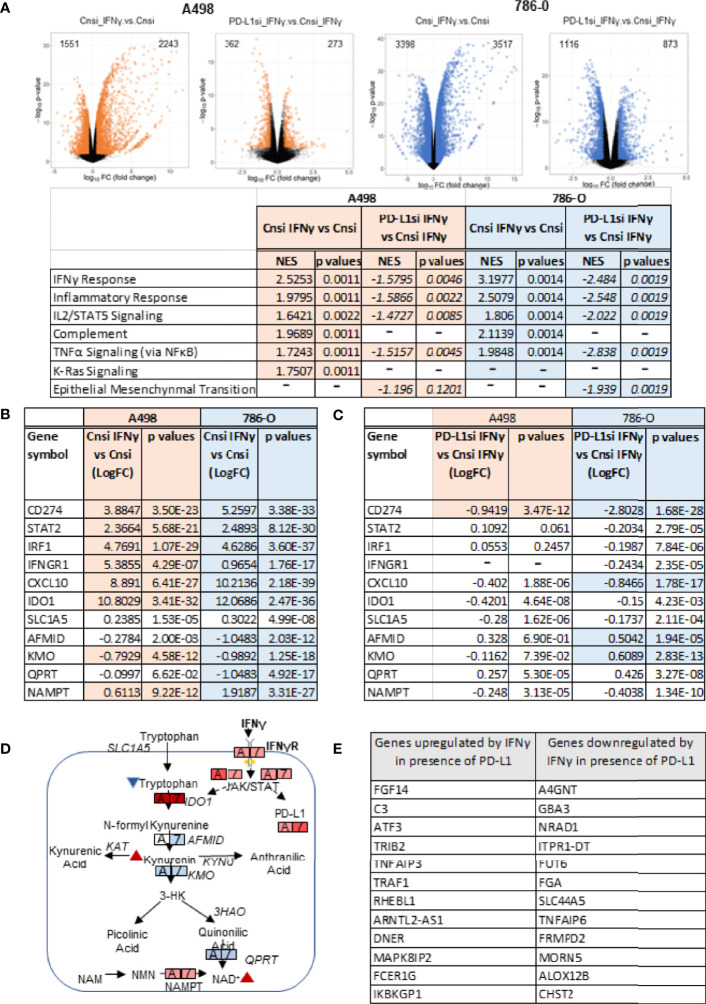
Transcriptional effects of IFNγ treatment with or without PD-L1 silencing. **(A)** Volcano plots depicting the number of genes upregulated and downregulated in A498 and 786-O cells with PD-L1 expression (Cnsi_IFNγ vs Cnsi) and with silenced PD-L1 expression (PD-L1si_IFNγ vs Cnsi_IFNγ) (n=3, for each group). The GSEA analysis indicating upregulated signaling pathways with IFNγ treatement in A498 and 786-O cells is shown in the table below the volcano plots, “-”, data not determined; RNA-Seq differential expression of genes (DEG) in A498 and 786-O cells with high PD-L1 expression (Cnsi_IFNγ vs Cnsi) are shown in **(B)** and with silenced PD-L1 expression (PD-L1si_IFNγ vs Cnsi_IFNγ) are shown in **(C)**, “-”, data not determined. **(D)** illustration of the effect of IFNγ on tryptophan metabolism; **(E)** Genes regulated by IFNγ in a PD-L1 dependent manner.

To evaluate the role played by PD-L1 in IFNγ signaling in ccRCC and to assess whether it might play a role in IFNγ-mediated regulation of cellular metabolism, we transiently silenced PD-L1 using a pooled small interference RNA on both A498 and 786-O cells, using scrambled RNA as controls. We confirmed the silencing efficiency by immunoblotting as shown in **Figure 3A** (see also **Supplemental Figures 2A, B**). While IFNγ (50 ng/mL) increased the expression of PD-L1 (Cn siRNA with IFNγ group), the inducible expression of PD-L1 was blocked with PD-L1 siRNA (PD-L1 siRNA with IFNγ group). PD-L2 protein expression was not altered by PD-L1 siRNA, neither were the inductions of STAT1 phosphorylation and STAT1 and Jak2 expression. Upon IFNγ exposure, cells lacking PD-L1 expression presented a lower ECAR compared with cells transfected with scrambled RNA, while their OCR were unchanged (**Figure 3B** and **Supplemental Figure 2C**). This suggest that PD-L1 expression is important for IFNγ induction of aerobic glycolysis. Additionally, the glucose and glutamine consumption of ccRCC cells were both partially abrogated in cells in which PD-L1 was transiently silenced (**Figures 3C, D**, respectively). Taken together, these data demonstrate that PD-L1 expression is required to mediate, at least in part, the intrinsic metabolic effect of IFNγ in 786-O and A498 cell lines. To help differentiate a cell intrinsic role of PD-L1 on cell metabolism in absence of ligand from a ligand induced effect on the tumor cells, 786-O and A498 cell lines were treated with three PD-L1 blocking therapeutic antibodies (avelumab, durvalumab and atezolizumab) and a purified ligand (PD-1-Fc; 0.1 ug/mL) and their metabolic functions were analysed using a seahorse bioanalyzer. As shown in **Supplementary Figure 2D**, in presence of IFNγ, avelumab, durvalumab and atezolizumab and PD-1-Fc did not significantly affect the cells’s ECAR. These data suggest that the metabolic effect observed is mediated by a cell-intrinsic role of PD-L1 that is not ligand-induced, or at least, not ligand-induced in this *in vitro* model. The mechanism underlying how PD-L1 expression regulates cellular metabolism is, however, unclear.We then performed RNA sequencing (RNA-Seq) of A498, and 786-O cell lines exposed to IFNγ with and without PD-L1 silencing. Differential gene expression and geneset enrichment analysis were performed as described in the *Methods* section (**Figure 4A**). The effect of IFNγ, by itself, was analyzed in both cell lines transiently transfected with a control siRNA. Consistent with other models, IFNγ induced multiple signaling pathways such as the IFNγ response, an inflammatory response and the TNFα signaling (**Figure 4A**). It induced the expression of members of the IFNγ signaling pathway, including PD-L1 (*CD274*), Indoleamine 2,3-dioxygenase (*IDO1*), *IRF1, IFRNGR1* and *CXCL10* in both cell lines (**Figure 4B**). The expression of the tryptophan importer *SLC1A5* was unchanged and the expression of members of the kynurenine pathway such as quinolate phosphoribosyltransferase (*QPRT*) and *AFMID* were decreased following IFNγ exposure in both A498 but not in 786-O cells (**Figure 4B**). The RNA expression of *IDO1*, *kynurenine 3-monooxygenase (KMO)*, and of the *nicotinamide phosphoribosyltransferase (NAMPT)*, which plays an important role in maintenance of NAD^+^ levels in cells, were however increased *(IDO1* and *NAMPT*) and decreased (*KMO*) following IFNγ exposure in both cells (**Figures 4B, D**). These results agree with a previous study in ccRCC that showed that when *KMO* is downregulated, there is a concomitant increase in NAMPT expression (39). These data also are consistent with IFNγ metabolic effects described in **Figure 2** on the modulation of metabolites of tryptophane metabolism. Interestingly, despite the observed effect of PD-L1 on glycolysis, the expression of the genes regulating glycolysis or glutamine metabolism were not modulated by PD-L1 silencing.

Next, we evaluated how PD-L1 silencing affected the response of the cells to IFNγ. As shown in **Figure 4A**, silencing of PD-L1 significantly dampened the overall transcriptional effect of IFNγ on ccRCC cells when comparing IFNγ-exposed samples that were transfected with either Cnsi (control siRNA) or PD-L1si (PD-L1 siRNA). Transient silencing of PD-L1 reversed, at least partially, the IFNγ response, the inflammatory response and TNFα signaling *via* NFκB (**Figure 4A**) in both cell lines, while it only minimally affected the expression of enzymes involved in the tryptophan/kynurenine pathway (**Figures 4B, C**). Because IFNγ-mediated phosphorylation of STAT1 and Jak2 expression were not affected by transient PD-L1 silencing (**Figure 3A**), our data suggest that the modulation of the effect of IFNy by PD-L1 was either downstream or independent of the STAT/Jak pathway. Then, we asked which transcripts expression may be necessary to mediate IFNγ signaling in A498 cells by subtracting transcripts that were communally regulated by IFNγ in cells expressing or not PD-L1 to transcripts that are differentially expressed following IFNγ exposure (**Figure 4E**). Expression of *FGF14*, *ATF3*, as well as of members of the TNFα pathway (*TNFAIP3, TRAF1*), the complement component *C3*, several enzymes involved in O-glycan biosynthesis (*A4GNT*), fucosyltransferase (*FUT6*) and sulfo-transferase (*CHST2*) appear to require PD-L1 expression in order to be regulated by IFNγ exposure in A498 cells. These data suggest that PD-L1 may be mediating the IFNγ effect on inflammation *via* TNFα signaling and C3. PD-L1 may also support a role of IFNγ on protein/enzyme cellular localization and stability by modulating the expression of enzymes regulating carbohydrate modification (40), which may explain IFNγ’s effect observed on glycolysis.

### Role of PD-L1 Modulation on Cytotoxic T Lymphocytes Activation and Metabolism

**Figure 5:**
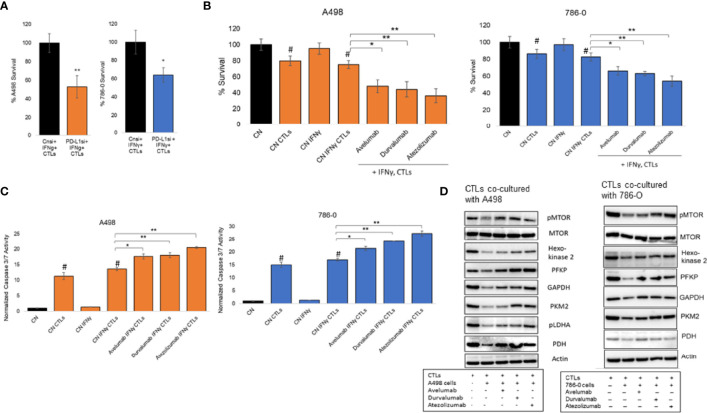
Modulation of PD-L1 in co-culture model. **(A)** Silencing of inducible expression of PD-L1 in A498 and 786-O in co-culture with CTLs decreases their viability. The data are presented as mean ± SD (n=3) *p<0.05, **p<0.001. **(B)** A498 and 786-O cells were co-cultured with CTLs in the presence absence of therapeutic monoclonal antibodies blocking PD-L1, avelumab, durvalumab and atezolizumab, at a concentration of 20 μg/ml for 24 hours (A498) or 48 hours (786-O). The data are presented as mean ± SD (n=3) *p<0.05, **p<0.001, ^#^p<0.05 for CN compared to CN CTLs (with and without IFNγ). **(C)** Caspase 3/7 activity was increased in A498 and 786-O cells co-cultured with CTLs in the presence of avelumab, durvalumab and atezolizumab at 20 μg/ml concentration for 24 hours (A498) and 48 hours (786-O). The data are presented as mean ± SD (n=3) *p<0.05, **p<0.001, ^#^p<0.05 for CN compared to CN CTLs (with and without IFNγ). **(D)** CTLs used for the co-culture experiments described above were then isolated to extract their protein content and analyse the expression of glycolytic enzymes, PDH and MTOR activity by immunoblotting (n=1). Densitometric analysis is shown in **Supplemental Figure 3**.

To examine the biological significance of increased expression of PD-L1 in ccRCC cancer immunity, we conducted co-culture experiments with cytotoxic T lymphocytes (CTLs). PD-L1 silencing and IFNγ treatment of A498 and 786-O ccRCC cells stably expressing Renilla luciferase were conducted as described in the *Methods* section. ccRCC cells were co-cultured with CTLs for 24 hours at a ratio of 1:3. Both A498 and 786-O ccRCC cells in which the inducible expression of PD-L1 was blocked, were more sensitive to CTLs cytotoxicity, compared to IFNγ treated cells alone (**Figure 5A**). Next, the effects of three monoclonal IgG1 antibodies targeting PD-L1 (avelumab, durvalumab and atezolizumab) were assessed in 786-O and A498 cell lines. The 2 cell lines were treated with IFNγ for 24 hours and then co-cultured with CTLs in the presence or absence of 20 μg/mL of avelumab, durvalumab and atezolizumab as indicated in the Figure legends. At the end of the experimental time, cytoxicity of CTLs was estimated by measuring Renilla luminescence, which gives a direct measure of number of live ccRCC cells. As shown in **Figure 5B**, avelumab, durvalumab, and atezolizumab significantly decreased the viability of ccRCC cells as compared to isotype control. In concordance with these results, these PD-L1 antibodies increased caspase 3/7 activity in both ccRCC cells when compared to their isotype control (**Figure 5C**), indicating induction of apoptosis in A498 and 786-O by CTLs in the presence of PD-L1 blockers. These data are consistent with the mechanism of action of this class of therapeutic agents and show that blocking PD1/PD-L1 interaction with avelumab, durvalumab and atezolizumab restored the cytotoxic activity of CTLs and promotes anti-tumor activity by inducing apoptosis in both A498 and 786-O ccRCC cells.

Given these observations, we investigated whether the change in CTLs activity correlated with an alteration of their metabolism. CTLs were isolated from co-culture experiments and protein expression of glycolytic enzymes and mTOR activity was assessed by immunoblotting. These enzymes included hexokinase2, phosphofructokinase (PFKP), glyceraldehyde 3-phosphate dehydrogenase (GAPDH), pyruvate kinase2 (PKM2), phosphorylated LDHA (pLDHA) and pyruvate dehydrogenase (PDH). Co-culture of CTLs with A498 and 786-O cell lines decreased mTOR phosphorylation and the expression of the glycolytic enzymes assessed, as compared to activated CTLs alone (**Figure 5D**, densitometry is in **Supplemental Figures 3A, B**, unprocessed blots in **Supplemental Figures 4**, **5**), suggesting that CTLs activation is inhibited when co-cultured with A498 and 786-O. The effect was reversed in the presence of the 3 PD-L1 blocking therapeutic antibodies avelumab, durvalumab, and atezolizumab. These results are in accordance with the current literature and suggest that the interaction of A498 and 786-O ccRCC cells with activated CTLs decreases the glycolytic capacities of CTLs, and therefore their activity, as compared to activated CTLs. Blocking the interaction between PD1/PD-L1 with avelumab, durvalumab and atezolizumab, restored the glycolytic metabolism of CTLs and their cytotoxicity.

## Discussion

Immune checkpoint inhibitor (ICI) therapies targeting programmed death receptor 1 (PD-1), programmed death receptor ligand 1 (PD-L1), and cytotoxic T lymphocytes antigen 4 (CTLA4) have become a major modality for the treatment of ccRCC (41, 42). However, their mechanism of action is not fully understood. In this nonclinical investigation, we demonstrated that PD-L1 expression was necessary to mediate some of the molecular effects of IFNγ on ccRCC cell lines (**Figure 4**). We also observed, as previously reported, that modulation of PD-L1, by silencing or with PD-L1 monoclonal antibodies, significantly induced CTLs mediated killing of ccRCC cells, A498 and 786-O. PD-L1 modulation altered the cellular metabolism of both CTLs and ccRCC cells, which may play a role in the observed CTLs cytotoxicity (**Figure 5**). IFNγ promoted a metabolic shift towards aerobic glycolysis in ccRCC cell lines *in vitro* (**Figures 1**, **2**). In the TME, metabolic interplay exists between tumor and immune cells, as activated T cells and tumor cells both rely on glycolysis for activation and survival and therefore compete for available resources. For example, lactate secreted by aerobic tumor cells and present in the TME has also been shown to suppress function, proliferation, and cytokine production of CTLs (43). In our study, metabolomic and extracellular flux analysis indicated that IFNγ exposure increased aerobic glycolysis in both A498 and 786-O cell lines (**Figures 1** and **2**). Silencing of inducible expression of PD-L1 in A498 and 786-O cells reduced ECAR, glucose and glutamine consumption, which would potentially make more glucose available for T cells in the TME (**Figure 3**). These results agree with previous reports indicating that PD-L1 has an intrinsic role on tumor metabolism, i.e., that PD-L1 has T cell independent functions (20, 25). In addition, PD-L1 was key in mediating IFNγ regulation of glucose and glutamine metabolism, although not through transcriptional regulation of metabolic enzymes (**Figures 3**, **4**) and unlike a report of PD-L1 regulating HK2 in non-small cell lung cancer (25). This difference might be tissue-type dependent. In our study, the mechanisms responsible for PD-L1 mediation of IFNγ’s cellular metabolic effect remain unclear. We have shown that PD-L1 expression was necessary for IFNγ-mediated regulation of ATF3, members of the TNFα pathway, as well as several enzymes involved in O-glycan biosynthesis and fuco- and sulfo-transferases. One possible explanation might be that PD-L1 plays a role in regulating enzyme/protein cellular localization and stability (40), which in turn, may affect the cells’ ability to consume glucose and glutamine.

The TME is a diverse and complex mixture consisting of malignant tumor cells, various infiltrating immune cells, fibroblasts and numerous cytokines and chemokines. The infiltrated immune cells in the TME play a crucial role in tumor growth, invasion, and metastasis (44). The anti-tumor functions of T cells are suppressed by various mechanisms (45–47), including by the interaction of PD-1 with PD-L1 (48). In this study, we reported that IFNγ contributes to immune evasion by upregulating the expression of PD-L1 in A498 and 786-O cells, likely through activation of the JAK/STAT signaling pathway. Chronic inflammation is known to enable tumor development by increasing genome instability, tumor cell growth, and angiogenesis (49). Differentially expressed genes and GSEA analysis revealed that overlapping pathways and genes including IFNγ response, inflammatory response, IL2 STAT5 signaling, complement, and TNFα signaling *via* NFkB related to inflammation were upregulated with IFNγ treatment in both A498 and 786-O cells (**Figure 4**). Silencing of PD-L1 significantly suppressed inflammatory pathways upregulated by IFNγ, suggesting that targeting PD-L1 could enhance tumor suppression in ccRCC. In addition, and consistent with the literature, blocking the interaction of PD-L1/PD1 with PD-L1 specific monoclonal IgG1 antibodies (avelumab, durvalumab, and atezolizumab) increased the cytotoxic effect of CTLs on ccRCC cells and modulated the cellular metabolism of CTLs (**Figure 5**). Previous studies have reported that activated CTLs depend on aerobic glycolysis for optimal T effector function (24, 50). Our data show that co-culture of A498 and 786-O cells with CTLs, downregulated glycolysis enzymes and mTOR activation, thus explaining the decrease of effector function of the CTLs. Co-culture of A498 and 786-O cells with CTLs in the presence of PD-L1 antibodies increased the expression of glycolysis enzymes and mTOR phosphorylation and restored their cytotoxic effector function.

Even though IFNγ signaling and PD-L1 have been tightly linked, our data and those of others demonstrate that IFNγ induces an intrinsic effect in tumor cells that is independent of PD-L1 expression. Silencing of PD-L1 did not affect IFNγ activation of STAT1 and Jak2 in 786-O and A498 cells (**Figure 3A**). These data are consistent with a recent article by Benci *et al.* that shows that an increase in the STAT1 signaling with IFNγ in the TME can trigger epigenetic and transcriptional changes of tumor cells and promote PD-L1 independent resistance to immune checkpoint therapy (51). STAT5 and STAT3 signaling can promote tumor progression by regulating the expression of cell cycle, survival and proinflammatory genes (52, 53). Activation of NF-κB and STAT is associated with cancer associated inflammation by secreting cytokines that stimulate tumor survival and clonogenic capacity (49, 54). The oncometabolite kynurenine has been shown to be secreted by tumor cells and to promote cancer associated inflammation (33). One-way IFNγ could exert immune suppression in ccRCC could be by increasing the level of kynurenine. We showed that IFNγ activates the tryptophane/kynurenine pathways both at the metabolite level (**Figure 2**) and at the transcriptional level (**Figure 4**) by upregulating of the expression of enzymes from the tryptophan pathway, including the tryptophan metabolizing enzyme indoleamin-2,3-dioxygenase (IDO1). Previous studies have shown that IDO1 and 2 can suppress anti-tumor immune response by activating CD4+CD25+Foxp3+ regulatory T cells (Treg) (14, 55–57) and myeloid-derived suppressor cells (MDSCs) (58), which consequently inactivate CTLs. Also, it has been reported that IFNγ can inactivate dendritic cells by inducing IDO1 expression (59, 60). Interestingly, silencing of PD-L1 had only a mild effect on IDO1 expression and did not revert the effects observed with IFNγ on the tryptophane/kynurenine pathway (**Figure 4**). These data suggest that IFNγ-induced tryptophane/kynurenine pathway may play a role in PD-L1 independent resistance to immune checkpoint therapy, and therefore, inhibition of this pathway might have a therapeutic value when combined with PD-L1 blocking therapies.

Together, our data demonstrate a central role for PD-L1 in mediating some of IFNγ metabolic and transcriptional effect in ccRCC cells and provides a rationale to further study how immunotherapies affect ccRCC metabolism and whether the efficacy of ICIs, including development of ICIs resistance in ccRCC, could be monitored by imaging ccRCC metabolism.

## Data Availability Statement

The datasets presented in this study can be found in online repositories. The names of the repository/repositories and accession number(s) can be found below: GEO database, accession GSE199107​​​​​​​.

## Author Contributions

Conceptualization, CS and MG. MG performed most of the experiments with the help from SG, SP and GL. Data analysis and interpretation, MG and CS. GSEA analysis, AN. Acquiring RNA-Seq data, C-KC, WW, and R-FS. Writing and editing original draft preparation, MG and CS. Supervision, CS. All authors have read and agreed to the published version of the manuscript.

## Funding

This project was entirely funded by US FDA intramural funds.

## Author Disclaimer

This article reflects the views of the authors and should not be construed to represent US FDA’s views or policies.

## Conflict of Interest

The authors declare that the research was conducted in the absence of any commercial or financial relationships that could be construed as a potential conflict of interest.

## Publisher’s Note

All claims expressed in this article are solely those of the authors and do not necessarily represent those of their affiliated organizations, or those of the publisher, the editors and the reviewers. Any product that may be evaluated in this article, or claim that may be made by its manufacturer, is not guaranteed or endorsed by the publisher.
