# Supplementary figures and images for: PD-L1 Mediates IFNγ-Regulation of Glucose but Not of Tryptophan Metabolism in Clear Cell Renal Cell Carcinoma
Source: Front Oncol. 2022 May 17;12:858379. doi: 10.3389/fonc.2022.858379 (PMC9152103; doi:10.3389/fonc.2022.858379)

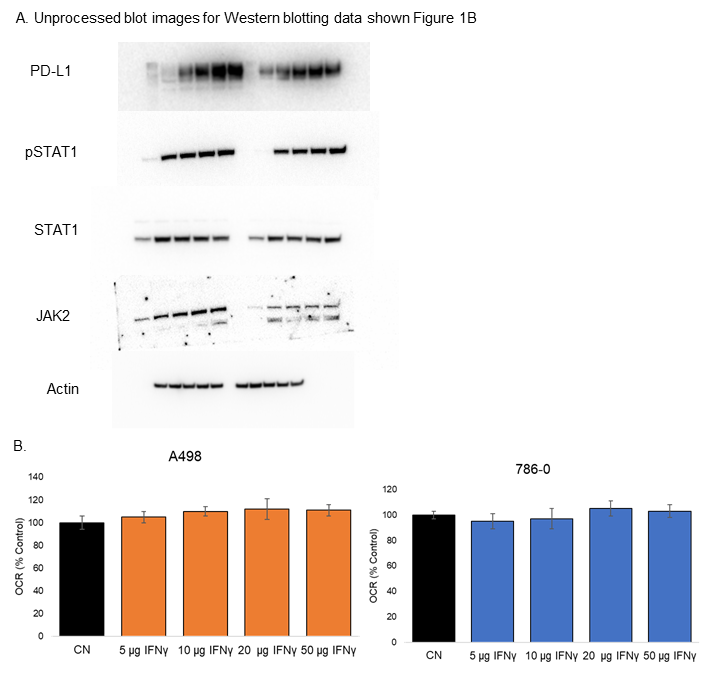

Supplement: Supplementary file 3 [file Image_1.tif]

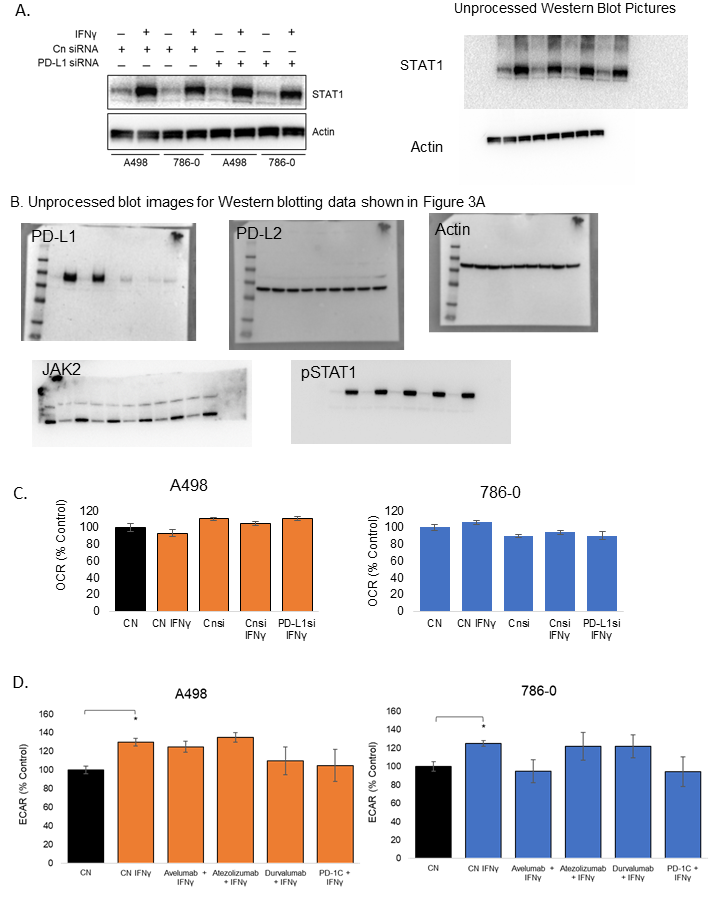

Supplement: Supplementary file 4 [file Image_2.tif]

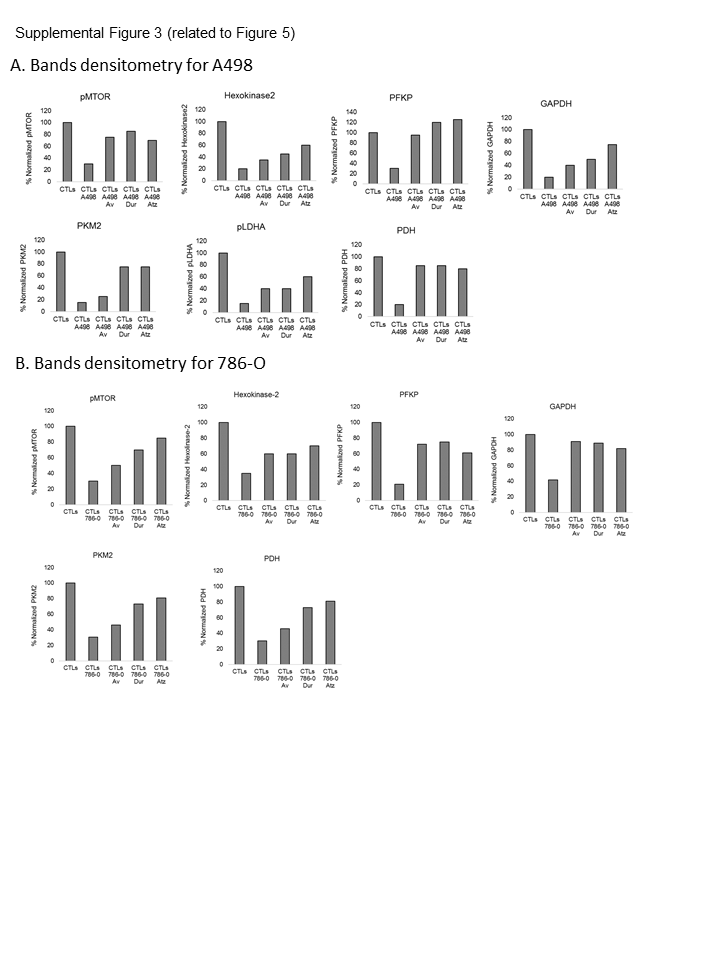

Supplement: Supplementary file 5 [file Image_3.tif]

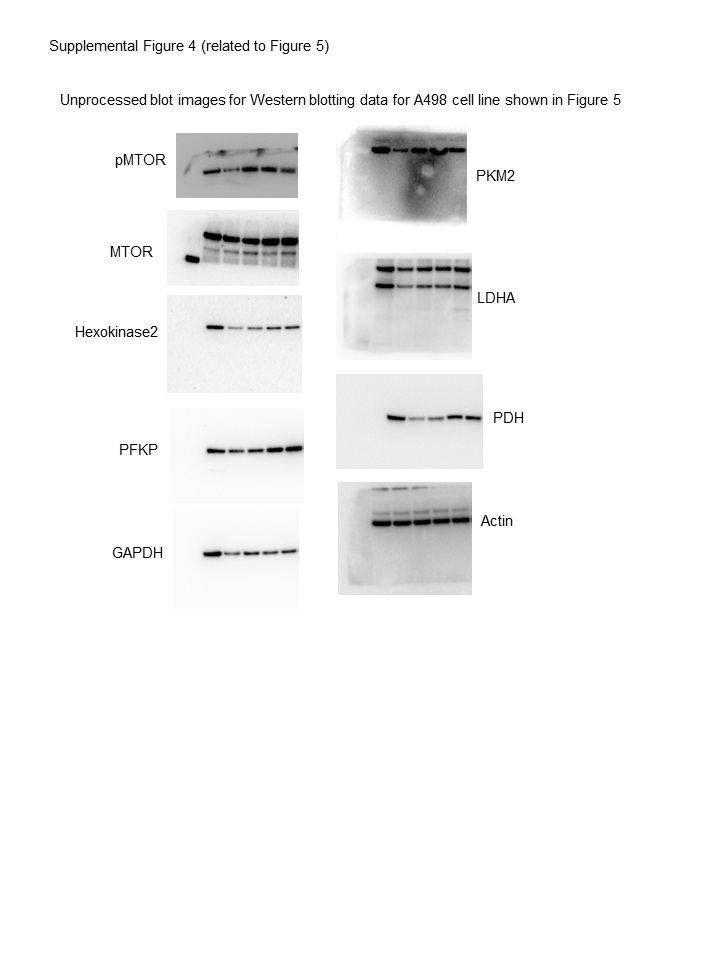

Supplement: Supplementary file 6 [file Image_4.tif]

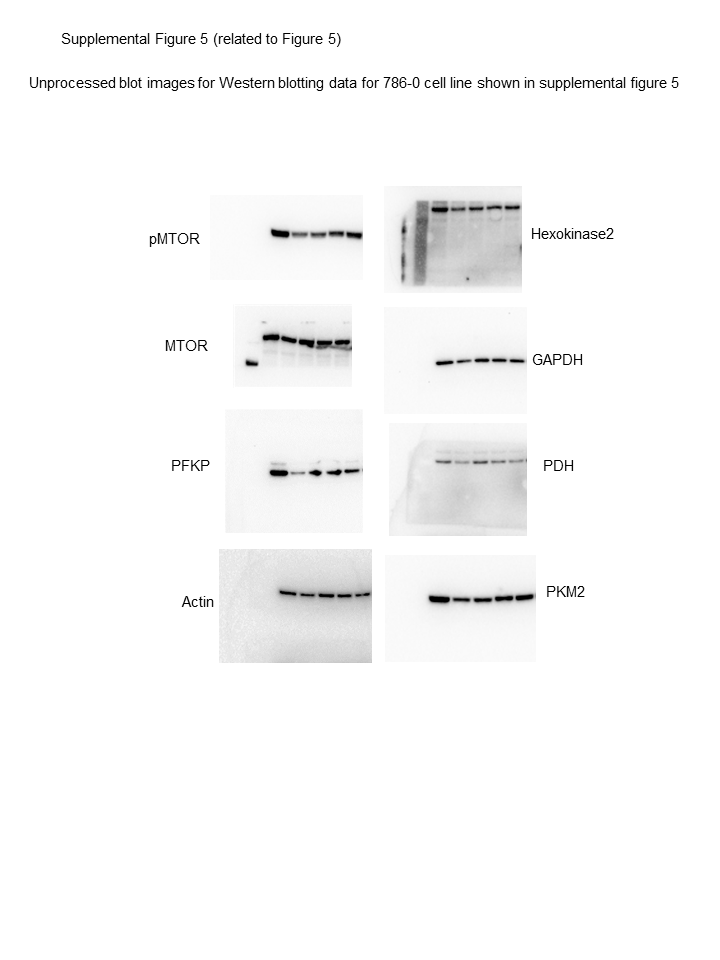

Supplement: Supplementary file 7 [file Image_5.tif]
